# School-Based Exercise Programs for Promoting Cardiorespiratory Fitness in Overweight and Obese Children Aged 6 to 10

**DOI:** 10.3390/children9091323

**Published:** 2022-08-30

**Authors:** Stefan Mijalković, Dušan Stanković, Mario Tomljanović, Maja Batez, Maki Grle, Ivana Grle, Ivan Brkljačić, Josip Jularić, Goran Sporiš, Suzana Žilič Fišer

**Affiliations:** 1 Faculty of Sport and Physical Education, University of Niš, 18000 Niš, Serbia; 2 Faculty of Kinesiology, University of Split, Ruđera Boškovića 31, 21000 Split, Croatia; 3 Faculty of Sport and Physical Education, University of Novi Sad, 21000 Novi Sad, Serbia; 4 Orthopedic Clinical Department, University Clinical Hospital in Mostar, 88000 Mostar, Bosnia and Herzegovina; 5 Clinical Department for Physical Medicine and Rehabilitation, University Clinical Hospital in Mostar, 88000 Mostar, Bosnia and Herzegovina; 6 Faculty of Kinesiology, University of Zagreb, 10000 Zagreb, Croatia; 7 Institute of Media Communications, Faculty of Electrical Engineering and Computer Science, University of Maribor, Koroška Cesta 46, 2000 Maribor, Slovenia

**Keywords:** physical activity, physical fitness, motor competence, children, health, monitoring and promoting, sport, sedentary behavior, obesity, well-being

## Abstract

The aim of this study was to conduct a systematic review of the school-based exercise programs for promoting cardiorespiratory fitness in overweight and obese children aged 6 to 10. Electronic databases (Web of Science and PubMed) were used as searching tools for collecting adequate studies published in the past 20 years. A total of 13 studies met the criteria for inclusion in this review, with a total of 2810 participants, both male and female. According to the results of this systematic review, overweight and obese children aged 6 to 10 who underwent certain interventions had their CRF improved. Furthermore, evidence suggested that interventions carried out during a longer period of time suggested led to greater improvement of cardiorespiratory fitness than a shorter one, but the level of cardiorespiratory fitness gradually decreases after the intervention.

## 1. Introduction

Cardiorespiratory fitness (CRF) is one of the most important health components of physical fitness [1], which is mainly expressed in maximal oxygen intake (VO2max) or in metabolic equivalents (MET) [2]. Cardiorespiratory fitness in children has a well-established link to overall health in youth and can lower the risk of cardiovascular disease (CVD) in later life [3,4,5]. Furthermore, it has been shown that there is a link between low CRF in childhood and early mortality in adulthood [6]. Therefore, in order to minimize the effects of CVD later in life and to prevent early mortality, it is strongly advised to concentrate on improving CRF from a young age [7,8]. It is well documented that overweight and obese children have lower CRF, and they are not able to train as hard and intensively as children with normal weight [9]. Furthermore, being overweight or obese as a child raises the risk of CVD in adulthood [10,11]. The negative effects of CRF and obesity from early age may be affected by being physically inactive [12,13]. On the other hand, it is proven that being physically active greatly influences the improvement of CRF in overweight and obese children aged 6 to 10 [14,15,16,17].

There is an increase in school interventions aiming to improve and promote CRF in early childhood [18,19,20,21,22]. Castro-Piñero et al. [23] state in their study that a CRF is a reliable indicator of CVD risk and should be tracked to identify children who may be at CVD risk. This study also suggests that, between baseline and follow-up, VO2max considerably decreased in both boys and girls (*p* < 0.001). They also concluded that the CRF should be a monitored system in order to prevent the potential occurrence of CVD. Regarding the program frequency, in order for overweight and obese children to have positive results, the CRF exercise program should be conducted three to four times a week for at least 6 weeks [24]. Recently, high-intensity circuit training (HIIT) has shown to be an effective exercise intervention that led to significant improvements and, therefore, could be included in regular classes [25]. Studies suggest that the HIIT method leads to a large improvement in CRF in children and affects the parameters related to neuromuscular and aerobic performance [26]. Furthermore, Stanly and Dharuman [27] state, in their study, that tai-chi, pilates, and yoga have proven to be methods that greatly influence the improvement of CRF.

It is essential to increase people’s understanding of how low CRF may have a range of negative effects throughout life [28], and its development should start in childhood. Therefore, improving CRF should be an integral part of physical education programs in all lower grades of primary schools. The aim of this work was to conduct a systematic review of the school-based exercise programs for promoting CRF in overweight and obese children aged 6 to 10.

## 2. Materials and Methods

### 2.1. Literature Identification

According to the PRISMA (Preferred Reporting Items for Systematic Reviews and Meta-Analyses) standards, studies were searched and analyzed [29]. The following databases were searched to collect relevant literature for this study: PubMed and Web of Science. The following terms were used during the search: ((school-based OR school-program OR intervention OR preschool OR primary school OR elementary school) AND (cardio-respiratory fitness OR CRF OR cardio fitness OR VO2max OR maximal oxygen consumption OR heart rate)) AND (overweight OR obese) NOT disease. Child: 6–12 years filter was turned on (Table 1). Studies are selected on the basis of titles, keywords, and abstracts, but primarily on the basis of the content of the study published in its entirety.

The data were analyzed using the descriptive approach, and the titles and abstracts evaluating CRF in overweight and obese children were used to determine whether or not a particular study was included. Studies were carefully identified, and they were only deemed pertinent if they fit the inclusion criteria. Two authors (D.S. and S.M.) carried out the research search, value evaluation, and data extraction. Each author then carried out cross-identification of studies, after which the study was either accepted or rejected for further analysis.

### 2.2. Inclusion Criteria

**Figure 1:**
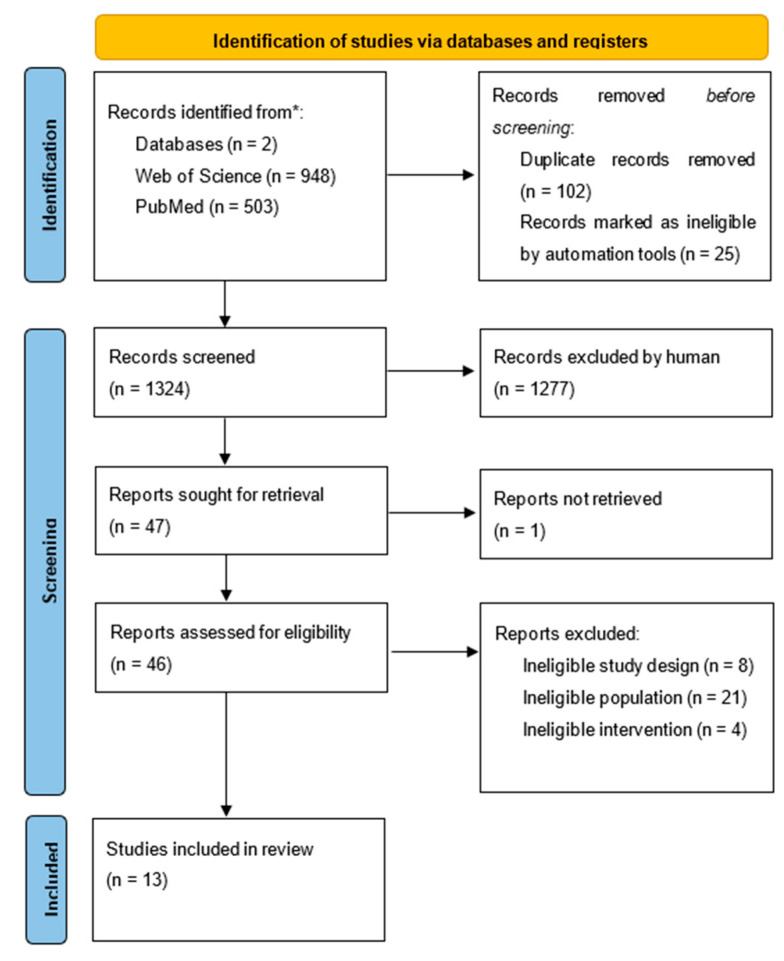
PRISMA flow diagram.

To be taken into account for the final analysis, the study had to meet the following criteria: The first requirement was that the study examines the relationship between school exercise programs and CRF among overweight and obese children aged 6 to 10. This selection criterion was used to rule out studies that included children who were not of this age and studies whose goal was not to determine how the school exercise program affected cardiorespiratory fitness. The second requirement was that the study’s participants had to be overweight or obese. The research had to have been published within the last 20 years, which was the third requirement. The fourth criterion was that the studies were published in English. The fifth criterion was that studies were original research (Figure 1).

### 2.3. Risk of Bias Assessment

The study’s quality and viability for inclusion, in the final analysis, were evaluated by two separate authors (S.M. and D.S.). The “Rayyan” web tool was used to do blind reviewing. A third reviewer (M.T.), who made the ultimate determination in cases of dispute on the findings on the assessment of the risk of bias, evaluated the collected data.

## 3. Results

### 3.1. Quality of the Studies

Pedro scale results were shown in Table 2. The total number of studies included in the quantitative synthesis and the points each study obtained on the PEDro scale were used to generate the study assessment scores [30]. The first criterion, which determines eligibility, is concerned with external validity but is not factored into the final result.

### 3.2. Selection and Characteristics of Studies

The electronic databases were searched, and 1451 studies were located. Following the elimination of duplicate research, systematic reviews, and meta-analyses, 1324 studies were left. After 1277 research were disqualified owing to inclusion requirements, 42 studies were evaluated for eligibility. A total of 13 papers were included in the final analysis after the remaining studies were reviewed and thoroughly read (Table 3).

Thirteen studies met the inclusion criteria for inclusion in this review. The oldest study was published in 2011 [31], and the most recent one is from 2022 [15]. The total number of participants was 2810. The highest number of participants was 574 [33] (Yin), and the lowest number of participants was 41 [14]. In almost all studies, the participants were both sexes. However, in two studies, the participants were only male [14,36], while no study was performed with females only. The longest intervention (36 months) was by [33], and the shortest interventions (two and a half months) were by Krustrup et al. [34] and Tan et al. [36]. All studies aimed to improve CRF, and post-intervention CRF improvement was found in all studies except in one [22]. The interventions most used in the studies were high-intensity interval training [15,19,37] and daily physical activity [16,32,33,35,36].

There were eight studies that increased VO_2max_/VO_2peak_ with physical activity sessions that lasted between 20 to 90 min; session frequency varied between daily, four times a week, two times a week, and once a week [15,16,17,19,32,35,36,37]. Heart rate and RHR were improved in three studies [14,31,33] by physical activity sessions that lasted either 20, 60, or 120 min; session frequencies varied between daily, two times a week, and three times a week. Blood pressure was improved in three studies [14,15,17] by physical activity sessions that lasted 20 or 60 min, and session frequencies varied between two, three, and four times a week.

## 4. Discussion

The current study aimed to conduct a systematic review of the school-based exercise programs for promoting cardiorespiratory fitness (CRF) in overweight and obese children aged 6 to 10. CRF has an important role in children’s health status. According to the results of the reviewed studies, twelve school-based programs have shown to affect the improvement of CRF in overweight and obese children aged 6 to 10 to some extent. Consequently, interventions such as high-intensity interval training, plyometric training, multidisciplinary weight reduction program (Kids4Fit), football, and active video gaming have a positive influence on CRF in children and reduce the risk of CVD. Therefore, the school’s physical education program should include exercises for promoting CRF in order to increase their aerobic capacity.

A key goal of lowering cardiovascular complications is to increase exercise capacity and CRF [38]. The main parameters that indicate CRF improvements tend to be VO_2max_, VO_2peak_, excess post-exercise oxygen consumption (EPOC), and resting HR [14,15,19,33,35,36,37]. Except for one study [22], all studies that have been reviewed achieved improvements in CRF with specific intervention. However, the question is whether these are safe and appropriate interventions for children aged 6 to 10 in terms of individualization and specificity [39]. Yin et al. [33], in their study, showed a positive trend in improving CRF, which is shown by heart rate (bpm) (*p* < 0.001), in favor of the intervention group. Additionally, plyometric training has proved to be another beneficial tool for lowering heart rate in a resting position [14]. Tan et al. [36] tend to improve CRF through various 40 min physical activities (walking, running, and ball games). A positive method in this study was that assessors had constant control over the subjects’ HRs which they wore during every training session. In another study, Thivel et al. [31] showed similar results according to CRF in the control and intervention groups. Actually, both control and intervention groups had significant increases in CRF, which was defined by the number of fully completed stages in the shuttle run.

There is strong evidence that high-intensity interval training (HIIT) can be a feasible and powerful tool for improving CRF [40,41,42,43]. The literature review reveals that HIIT training is popular among older populations [44,45,46,47]; however, there are papers that integrate school-based programs for younger groups aged 6 to 10. Martinez-Visciano et al. [15] concluded that HIIT training improved the girls’ CRF throughout one school year. On the other hand, Martinez et al. [19] proved that VO_2max_ in overweight children was enhanced in only three months while conducting two HIIT training sessions per week and using high-intensity intermittent exercises and sports activities such as: half-squats, sprints, jumps, and horizontal shot puts. In addition, strategies with exercise machines such as bicycles and treadmills, as well as basic motor skills (running, jumping, throwing), were applied in high-intensity programs [37]. Evidence suggests that school-based HIIT training program leads to improvement of aerobic capacity of overweight and obese children.

School-based exercise programs for increasing CRF in overweight and obese children aged 6 to 10 included after-school aerobic workouts, moderate to vigorous physical activity (MVPA), the multidisciplinary weight reduction program (Kids4Fit), and the intensity of maximal fat oxidation rate (FATmax) [16,32,33,35,36]. It can be said that CRF and aerobic capacity of children aged 6 to 10 have significantly improved as a result of everyday participation in these physical activities. Additionally, Leeuwen et al. [17] used Kids4Fit as an intervention to enhance CRF, with the intervention taking place twice a week during the initial six weeks while being carried out the intervention once a week during the final six weeks. While conducting this study, a significant positive effect on CRF was also noticed in overweight and obese children, but after the intervention, CRF gradually declined. These findings would suggest that Kids4Fit was a good school-based intervention program for promoting CRF in children aged 6 to 10, but it is necessary to do the intervention daily and for a longer period of time for the CRF to continue improving. The regular classes of physical education are not enough in order to promote the children’s CRF. However, if two additional workouts, which include exercises to improve coordination, strength, endurance, speed, and flexibility, are added to regular classes, the improvement of the CRF in overweight and obese children can be greatly influenced [31]. Furthermore, exergaming and small-sided football are two interesting and fun ways to include additional classes of vigorous intensity. In addition to children’s enjoyment, they also regulate their CRF by using these interventions [22,45]. It is necessary for the realization of children’s regular classes to be fun and playable in order to improve their CRF.

The main limitation of this review was the small number of articles engaging CRF in children aged 6 to 10. In addition, the majority of studies included children with average body weight. Secondly, the study covered only overweight and obese children who are more likely to increase their CRF due to them being less active than children of average weight. The third limitation of the study is the variety of ethnicities among participants since the mentalities of different cultures regarding physical activity and motivation differences.

## 5. Conclusions

The results of this systematic review showed that there were interventions that led to improvement in CRF in overweight and obese children aged 6 to 10. Long-lasting interventions led to greater improvement of CRF than a shorter intervention. Our findings provide evidence that school-based exercise programs greatly influence the CRF parameters such as maximal oxygen intake, peak oxygen uptake, heart rate, and resting heart rate, but it gradually declines after the intervention.

## Figures and Tables

**Table 1:** Search strategy to identify articles.

Search 1	Search 2	Search 3	Filters
school-based school program Intervention preschool primary school elementary school	cardio-respiratory fitness CRF cardio fitness VO2max maximal oxygen consumption heart rate	overweight obese	child: 6–12 years

**Table 2:** PEDro scale results.

	Criterion											
Study	1	2	3	4	5	6	7	8	9	10	11	∑
Thivel et al. (2011) [31]	Y	Y	Y	Y	N	N	N	Y	Y	Y	Y	7
Resaland et al. (2011) [32]	Y	N	N	Y	N	N	N	N	Y	Y	Y	4
Yin et al. (2012) [33]	Y	Y	Y	Y	N	N	N	N	Y	Y	Y	6
Krustrup et al. (2014) [34]	Y	Y	Y	Y	N	N	Y	Y	Y	Y	Y	8
Khan et al. (2014) [35]	Y	Y	Y	Y	Y	N	N	Y	Y	Y	Y	8
Tan et al. (2015) [36]	Y	Y	Y	Y	N	N	N	Y	Y	Y	Y	7
Martinez et al. (2016) [19]	Y	Y	Y	Y	N	N	N	Y	Y	Y	Y	7
Leeuwen et al. (2018) [17]	Y	N	N	N	N	N	N	Y	Y	N	Y	3
Ye et al. (2019) [22]	Y	Y	Y	N	N	N	N	Y	N	Y	Y	5
Davis et al. (2019) [16]	Y	Y	Y	Y	Y	N	N	Y	Y	Y	Y	8
Espinoza-Silva et al. (2019) [37]	Y	N	N	Y	N	N	N	Y	Y	Y	Y	5
Leandro et al. (2021) [14]	Y	Y	Y	Y	N	N	N	Y	Y	Y	Y	7
Martinez-Viscaiano et al. (2022) [15]	Y	Y	Y	Y	Y	Y	Y	Y	N	Y	Y	9

Legend: 1—eligibility criteria; 2—random allocation; 3—concealed allocation; 4—baseline comparability; 5—blind subject; 6—blind clinician; 7—blind assessor; 8—adequate follow-up; 9—intention-to-treat analysis; 10—between-group analysis; 11—point estimates and variability; Y—criterion is satisfied; N—criterion is not satisfied; ∑—total awarded points.

**Table 3:** Participants, variables, interventions and results of included studies.

First Author and Year of Publication	Sample of Participants		PF	Type of Intervention	Duration of Intervention	Results
	Number	Age			Weeks	
Thivel et al. (2011) [31]	N—457	6–10	SRT, CPP, RHR, HRR	AL, 2/week (60 min)	26	HRR↑ RHR↑ (E&C)
Resaland et al. (2011) [32]	N—256 M—125 F—131	9–10	TRE, VO_2peak_, HR_peak_	Daily, MVPA (60 min)	104	VO_2peak_↑ (E)
Yin et al. (2012) [33]	N—574	8.7 ± 0.5	ST, HR	Daily, Kids4Fit (120 min)	156	HR↑ (E&C)
Krustrup et al. (2014) [34]	N—51 M—21 F—30	9–10	CTE, IVRTglobal, LVSEF, LVPWD, RHR, RBP, HR, HR_max_	SSF, 3x/week (40 min)	10	LVSEF↑ IVRTglobal↑(E)
Khan et al. (2014) [35]	N—220 M—117 F—103	8–9	TRE, HR, VO_2max_	Daily MVPA (70 min)	39	VO_2max_↑ (E)
Tan et al. (2015) [36]	M—46	8–10	SRT, VO_2max_, HR, FAT_max_	Daily PA (40 min)	10	VO_2max_↑ (E)
Martinez et al. (2016) [19]	N—94 M—52 F-42	7–9	SRT, TRE, VO_2max_, EPOC, HR_max_	HIIT, 2x/week (90 min)	13	VO_2max_↑ EPOC↑ (E)
Leeuwen et al. (2018) [17]	N—154 M—66 F—88	8.5 ± 1.8	SRT, VO_2max_, BP	Kids4Fit, 2xweek; 1xweek (20 min)	13	VO_2max_↑ BP↑ (E)
Ye et al. (2019) [22]	N—81 M—42 F—39	9.23 ± 0.62	HMR, VO_2max_	EXG, 1/week (50 min)	35	/
Davis et al. (2019) [16]	N—75 M—29 F—46	9.5–9.8	TRE, PWV, BP, VO_2peak_	Daily, ASAE (40 min)	35	VO_2peak_↑ (E)
Espinoza-Silva et al. (2019) [37]	N—274 M—120 F—154	7–9	6MWT, BP, VO_2max_	HIIT, 2x/week (40–50 min)	30	VO_2max_↑ (E)
Leandro et al. (2021) [14]	M—41	7–9	ABPM, RHR, BP	PLT 3x/week (20 min)	13	BP↑ RHR↑ (E)
Martinez-Viscaiano et al. (2022) [15]	N—487 M—233 F—254	9.89 ± 0.71	SRT, BP, VO_2max_	HIIT, 4x/week (60 min)	39	BP↑ VO_2max_↑ (E/onlyF)

Legend: ↑ significant improvement; N—number of respondents; M—male participants; F—female participanrts; E—experimental group; C—control group; PF—physical fitness test; PA—physical activity; SSF—small-sided football; AL—additional lessons; PLT—plyometric training; HIIT—high intensity interval training; VO_2max_—maximal oxygen consumption; VO_2peak_—peak oxygen uptake; HR—heart rate; RHR—rest heart rate; HRR—heart rate reserve; HR_peak_—peak heart rate; HR_max_—maximum heart rate; LVSEF—left ventricular systolic ejection fraction; IVRTglobal—global isovolumetric relaxation time; LVPWD—left ventricular posterior wall diameter; PWV—Carotid-femoral pulse wave velocity; BP—blood pressure; RBP—rest blood pressure; EPOC—excess post-exercise oxygen consumption; ST—step test; 6MWT—6 min walk test; HMR—half-mile run; Kids4Fit—multidisciplinary weight reduction program; CTE—comprehensive transthoracic echocardiography; ABPM—automatic arterial blood pressure monitor; TRE—treadmill protocol; CPP—cycle peak power; SRT—shuttle run test; MVPA—moderate-to-vigorous intensity physical activity; EXG—exergaming; ASAE—after school aerobic exercise; FATmax—the intensity of maximal fat oxidation rate.

## Data Availability

Not applicable.
